# The value of observational registry studies for the next infectious disease emergency

**DOI:** 10.1128/mbio.02565-23

**Published:** 2023-11-08

**Authors:** Jonathon W. Senefeld, Peter Marks, Arturo Casadevall, Michael J. Joyner

**Affiliations:** 1 Department of Kinesiology and Community Health, University of Illinois at Urbana-Champaign, Urbana, Illinois, USA; 2 Center for Biologics Evaluation and Research, U.S. Food and Drug Administration, Silver Spring, Maryland, USA; 3 Department of Molecular Microbiology and Immunology, Johns Hopkins Bloomberg School of Public Health, Baltimore, Maryland, USA; 4 Department of Anesthesiology and Perioperative Medicine, Mayo Clinic, Rochester, Minnesota, USA

**Keywords:** coronavirus, neutralizing antibodies, clinical trials

## Abstract

During infectious disease emergencies, it may be necessary to deploy new therapies without conclusive evidence for their effectiveness. During the SARS-CoV-2 pandemic, several countries used registries to track the use of COVID-19 convalescent plasma (CCP). Those registries provided evidence that CCP was effective when used early and with high titer.

## EDITORIAL

The coronavirus disease 2019 (COVID-19) pandemic was associated with numerous barriers to clinical research and fundamentally challenged clinical research practices (1, 2). The COVID-19 pandemic placed incredible pressure on researchers, regulators, and policymakers to generate evidence-based recommendations for therapeutics; however, an overwhelmingly large number of clinical trials with questionable methodological quality were registered and executed (3). Thus, a more contemporary focus of clinical research is to use lessons learned during the COVID-19 pandemic to create a more effective environment to conduct clinical research. In this framework, we discuss how lessons learned from the COVID-19 pandemic can inform clinical research of antibody-based therapy with a key focus on both the utility of registry studies and the biological plausibility of antibody therapy.

In this edition of *mBio*, Perichon and colleagues report registry data associated with the early stages of the COVID-19 pandemic (1 June to 31 October 2020) in Argentina showing that the administration of high titer convalescent plasma (CP) earlier in the course of hospitalization was associated with better clinical outcomes (4). These results are similar to those reported for CP efficacy by registries in the United States (5), Italy (6), and another Argentinean registry (7). These findings are consistent with one of the big overarching lessons relearned during the COVID-19 pandemic, namely, that antibody therapy works but only when used properly (8). More specifically, the optimal use of antibody therapy includes treating outpatients early in the course of infection using high levels of specific antibodies to prevent hospitalizations and deaths (9, 10). Furthermore, antibody therapy reduces the risk of death among inpatients when patients are treated early in the course of the disease and outside of the intensive care unit (11). These two lessons were well known in the pre-antibiotic era but were forgotten and had to be painfully relearned during the COVID-19 pandemic (12, 13). In addition, among immunocompromised patients—who are unable to generate sufficient endogenous antibodies in response to infection—antibody therapy can work at any time during the course of the disease, and this was a discovery associated with antibody therapy during the recent COVID-19 pandemic (14–16). Beneath the overarching lesson are three caveats that should inform the use of antibody therapy in general and especially how CP is used and studied in the next pandemic.

### First caveat

The first caveat is that CP from recovered patients is likely to be the first form deployable of antibody therapy “next time”—during the next pandemic or infectious disease emergency. Given the safety profile of CP and the mortality benefit associated with CP during the COVID-19 pandemic (17, 18), early use of CP will almost certainly be considered the “next time” humanity faces an infectious disease emergency. CP may also be a durable therapeutic option because it is relatively inexpensive, available worldwide once there are survivors of the disease and can evolve as pathogen variants evolve (19). When vaccines become available, CP obtained from donors with hybrid immunity may be especially useful in immunocompromised patients (20, 21).

### Second caveat

The second caveat is that while CP will almost certainly be available early during the next crisis, there may be challenges with assaying and standardizing the amount and quality of the antibodies it contains (22). Assay systems to measure specific antibodies against new pathogens need time to be developed and deployed. High priority needs to be given to the rapid development of assays to determine viral neutralization capacity or antibody levels in CP. Without such assays, the dose of CP to give must be entirely empiric. Indeed, in the early days of CP usage during the COVID-19 pandemic, there were no commercial neutralization assay or assays for antibodies to SARS-CoV-2 and as many as one-third of CP units had little or no antibody. That said, given the relative safety of CP, which is essentially fresh frozen plasma with a high titer of antibody to a specific pathogen, this limitation should not necessarily stop early deployment of CP in the setting of an epidemic or pandemic that is taking people’s lives. In the absence of information about CP titers, physicians can hedge their bets that the patient is receiving a therapeutic dose using more than one unit from different patients. If CP is used at scale as part of simple but well-designed registries, insights into efficacy, timing, and dosing can be obtained quickly and inform its optimal use. Systematic storage of CP samples would allow them to be tested post hoc after the assay systems catch up. This would provide data on how antibody dose affects outcomes. In conjunction with data on the timing of the CP administration, de facto randomized and blinded data could be obtained on the optimal use case. Importantly, such data from CP registries can then inform the design of subsequent “definitive” trials of CP and other forms of antibody therapy.

### Third caveat

The third caveat is that the fundamentals of antibody therapy—which were well established before World War II and which were rediscovered during the SARS-CoV-2 pandemic—should not be forgotten again. Namely, for antibody therapy to work enough specific antibodies should be given early in the course of infection. This is not surprising since in the case of COVID-19 CP worked as an antiviral and, like small molecule antivirals, its efficacy is in viral neutralization and cannot be expected to reverse the pulmonary inflammatory damage that leads to hypoxia and death (8). This lesson was forgotten early in the COVID-19 pandemic and unfortunately led to treatment trials of CP late in the course of patients who were almost certainly too sick to benefit from antibody therapy. Unfortunately, the results of these trials then confused the emerging narrative about the efficacy and optimal use case for CP, causing a sense of disappointment that led many to abandon this therapy at a time when there were no alternatives (8). Importantly, starting randomized clinical trials (RCTs) without clear data on dose (which depends on titer, as noted above) and timing is a prescription for repeating the mistakes made when CP was studied in large high-profile RCTs like RECOVERY (23). A technically “well-done” trial is not well done when the wrong use case is tested making use of a product that is not appropriately defined.

When the big lesson and three caveats about antibody therapy in general and CP in specific are considered as a whole, a flexible pre-planned approach to early registry-based data collection could be lifesaving during the next infectious disease emergency. To do this, the approach of the U.S. Expanded Access Program for CP and the U.S. Convalescent Plasma Project studies that flowed from it can be refined and serve as a model for the needed infrastructure program (5):

1. The focus of treatment with CP in any infectious disease emergency should be early after symptoms arise—preferably in outpatients or immediately on admission to the hospital.
2. Regulators and funding agencies should be involved in the pre-planning and development of simple CP administration protocols and their oversight. They should also work to expedite the development and deployment of assays to determine the neutralization capacity or antibody content of CP.
3. Mechanisms including funding should be in place so that the blood collection and banking system can obtain CP from qualified recovered donors as soon as they become available. The experience with CP for COVID-19 suggests that local plasma is more likely to be effective than distantly source plasma (24). Samples of the CP should be stored via a central biobank for later analysis as assays come online.
4. A diverse network of sites should be available for the administration of CP with pre-established ground rules for a central IRB, patient consent, and the reporting of fundamental patient outcomes.
5. A data management system and analytics team should be in place to facilitate rapid reporting of results.
6. Simulation exercises should be performed to test the system.
7. All elements of the program should focus on simplicity, and the “enemy of good is perfect*...”* axiom of crisis-based medical care must be rigorously adhered to.

During the next pandemic, there will be an impetus to test CP in RCTs, but such trials should not commence until there is information on dosage and optimal timing of antibody therapy since it is impossible to design good studies without that knowledge (25). Information on optimal dose and timing can come from registries. Furthermore, given a safety record extending over more than one century, the deployment of CP should not be delayed since it is likely to be the only therapy available in the early days of a future emergency. The argument that deployment of CP without RCT data will preclude the completion of such trials was refuted by the experience in the United States where the CONTAIN trial was completed even when CP was available under Emergency Use Authorization (26). During 2020, the early administration of high titer convalescent plasma to a large number of hospitalized patients in the United States saved lives prior to the advent of vaccination and other therapies. The widespread use of CP was informed by simple registry-based data that were confirmed by retrospective cohort studies and later RCTs. In this context, the registry-based study of Perichon and colleagues conducted in Argentina and reported in this edition of *mBio* adds credence to the approach used in 2020 (4). The overall experience from that time also provides a road map for the lifesaving use of CP “next time.”
